# Two Faces of the Same Thrombus: Molecular Phenotypes of Thromboinflammation in Acute Pulmonary Embolism and the Path to Phenotype-Guided Risk Stratification

**DOI:** 10.3390/ijms27209260

**Published:** 2026-10-19

**Authors:** Hugo Hyung Bok Yoo

**Affiliations:** Pulmonary Division, Botucatu Medicine School, São Paulo State University (UNESP), Botucatu 18618-687, SP, Brazil; hugo.yoo@unesp.br

**Keywords:** acute pulmonary embolism, biomarkers, thromboinflammation, molecular phenotypes, neutrophil extracellular traps, complement, endothelial dysfunction, right ventricular failure, risk stratification

## Abstract

Acute pulmonary embolism (PE) is a potentially fatal cardiovascular emergency where early risk assessment determines treatment escalation. Clinical scores like PESI and sPESI are useful but capture only a single moment and cannot track evolving biological processes: coagulation on inflamed endothelium, nitric oxide signaling loss, and right ventricular pressure overload. Circulating biomarkers provide direct readouts of these events. This review proposes that acute PE molecular pathophysiology organizes into two overlapping phenotypes—two faces of the same thrombus. The first is a predominantly thromboinflammatory phenotype, driven by neutrophil extracellular traps (NETs), complement activation, and endothelial injury; the second is a predominantly myocardial phenotype, driven by right ventricular pressure overload and subendocardial ischemia. We examine each phenotype’s molecular basis and appraise how far corresponding biomarkers—D-dimer, cardiac troponins, natriuretic peptides, GDF-15, H-FABP, sST2, complement activation products, and inflammatory indices—have moved into clinical practice. Two issues deserve attention: biomarker kinetics during the first 72 h after symptom onset, and whether intermediate-risk patients can be separated into meaningful molecular phenotypes. We address implementation barriers: lack of harmonized assays, undefined decision thresholds, and absence of prospective multicenter validation. This narrative review connects disease mechanisms to which patients are most likely to deteriorate and outlines a research agenda for phenotype-guided risk stratification.

## 1. Introduction

Acute pulmonary embolism (PE) occurs when thrombi, usually originating in the deep veins of the lower limb, obstruct part of the pulmonary arterial bed. It represents a significant and growing cardiovascular burden worldwide, with recent nationwide data documenting a rising incidence and substantial in-hospital mortality [1]. The clinical presentation ranges from an incidental filling defect on imaging to sudden cardiovascular collapse, and this broad spectrum is why risk assessment, not diagnosis alone, drives management.

Clinical presentation ranges from an incidental filling defect on imaging to sudden cardiovascular collapse, and this broad spectrum is why risk assessment, not diagnosis alone, drives management. Computed tomography pulmonary angiography (CTPA) is the main confirmatory test, but it requires iodinated contrast, exposes patients to ionizing radiation, and is not universally available [2]. The Pulmonary Embolism Severity Index (PESI) and its simplified version (sPESI) are validated prognostic tools, but they summarize only a single point in time and do not directly reflect coagulation activity, inflammation, or myocardial injury. Their principal strength is identifying low-risk patients who may be suitable for outpatient care; their principal limitation is poorer discrimination in the intermediate-risk range, where the most difficult decisions are made [3].

Biomarkers are valuable because they provide insight into the molecular processes underlying disease progression rather than merely reflecting their clinical consequences. Several recent reviews have addressed the topic from a practical standpoint, focusing on individual markers or on the overall approach to risk stratification and treatment [4,5,6]. What these reviews address less comprehensively is the relationship between individual markers and the underlying molecular pathophysiology, and the extent to which this relationship explains clinical deterioration in some patients.

The present narrative review fills this gap in three specific ways. First, it develops a unified mechanistic framework organized around two overlapping molecular phenotypes: a predominantly thromboinflammatory phenotype driven by NETs, complement activation, and endothelial injury, and a predominantly myocardial phenotype driven by right ventricular pressure overload and subendocardial ischemia, so that each biomarker is understood as a readout of one or both phenotypes rather than as an isolated measurement. We deliberately frame these as two faces of the same thrombus to emphasize that they coexist and interact, rather than representing alternative forms of disease. Second, the framework is used to interpret the still understudied kinetics of biomarker release over the first 72 h after symptom onset, a temporal window in which serial rather than single measurements may prove decisive. Third, and most importantly, we ask whether intermediate-risk patients, where prognostic uncertainty is greatest, can be separated into distinct molecular phenotypes that might eventually guide phenotype-specific escalation of therapy.

Whereas earlier reviews catalogue markers, the present review asks which biological process each marker reports, when its signal becomes informative during the first days of disease, and in whom the signal should prompt escalation. Finally, the review identifies the specific barriers, assay harmonization, undefined thresholds, and the absence of prospective validation, that must be overcome before these markers can change management.

## 2. Literature Search Strategy

A literature search was conducted to identify publications relevant to the molecular pathophysiology of acute pulmonary embolism and the role of circulating biomarkers in risk stratification. Searches were performed in PubMed/MEDLINE, Web of Science, and Scopus, covering publications from January 2000 through August 2026. The search strategy combined Medical Subject Headings (MeSH) terms and free-text keywords related to pulmonary embolism, biomarkers, thromboinflammation, neutrophil extracellular traps, complement activation, endothelial dysfunction, right ventricular dysfunction, and specific biomarkers discussed in this review (troponin, natriuretic peptides, GDF-15, H-FABP, sST2, microRNAs, and inflammatory indices).

This article is a narrative review, not a systematic review. The objective was to synthesize mechanistic understanding and clinical evidence connecting molecular pathways to biomarker interpretation and risk stratification, rather than to conduct an exhaustive, protocol-driven assessment of all eligible studies. Accordingly, study selection was guided by relevance to the review’s central aims: explaining the biological basis of each biomarker, interpreting biomarker kinetics during the first 72 h after symptom onset, and evaluating whether intermediate-risk patients can be separated into distinct molecular phenotypes.

Priority was given to original human studies with defined clinical outcomes, mechanistic studies that informed the proposed thromboinflammatory and myocardial framework, systematic reviews and meta-analyses, and current society guidelines. Animal studies were included when they provided mechanistic evidence relevant to the pathways discussed. Studies were selected based on methodological clarity, clinical relevance, and independent replication where available. Case reports, conference abstracts without subsequent full-text publication, editorials, and studies with insufficient methodological detail were not used as principal evidence. Reference lists of relevant reviews and key original articles were also screened to identify additional pertinent publications not captured by the database searches.

Because this is a narrative review and no formal systematic screening process with stage-by-stage counts was conducted, a PRISMA flow diagram is not reported. The selection process was strictly guided by the overarching aim of connecting molecular pathophysiology to clinical risk stratification and by the need to provide a coherent, mechanism-based interpretation of biomarker findings.

## 3. Molecular Pathophysiology

### 3.1. Thromboinflammation

For many years, acute PE was treated as a purely mechanical problem, under the assumption that removing the obstruction would simply restore normal physiology. Two clinical observations challenge this view. First, patients with a similar clot burden can exhibit strikingly different outcomes. Second, some patients continue to deteriorate even after what appears to be a successful reperfusion [5,6]. These discrepancies suggest that factors beyond the mechanical obstruction contribute to disease progression.

A growing body of evidence implicates the inflammatory response as a key determinant: reperfusion itself can trigger a systemic and local inflammatory cascade, and the magnitude of this response varies considerably among patients, influencing both recovery and the risk of secondary deterioration. This has shifted attention toward the interplay between coagulation and immunity, a relationship increasingly recognized under the term thromboinflammation [7].

**Figure 1:**
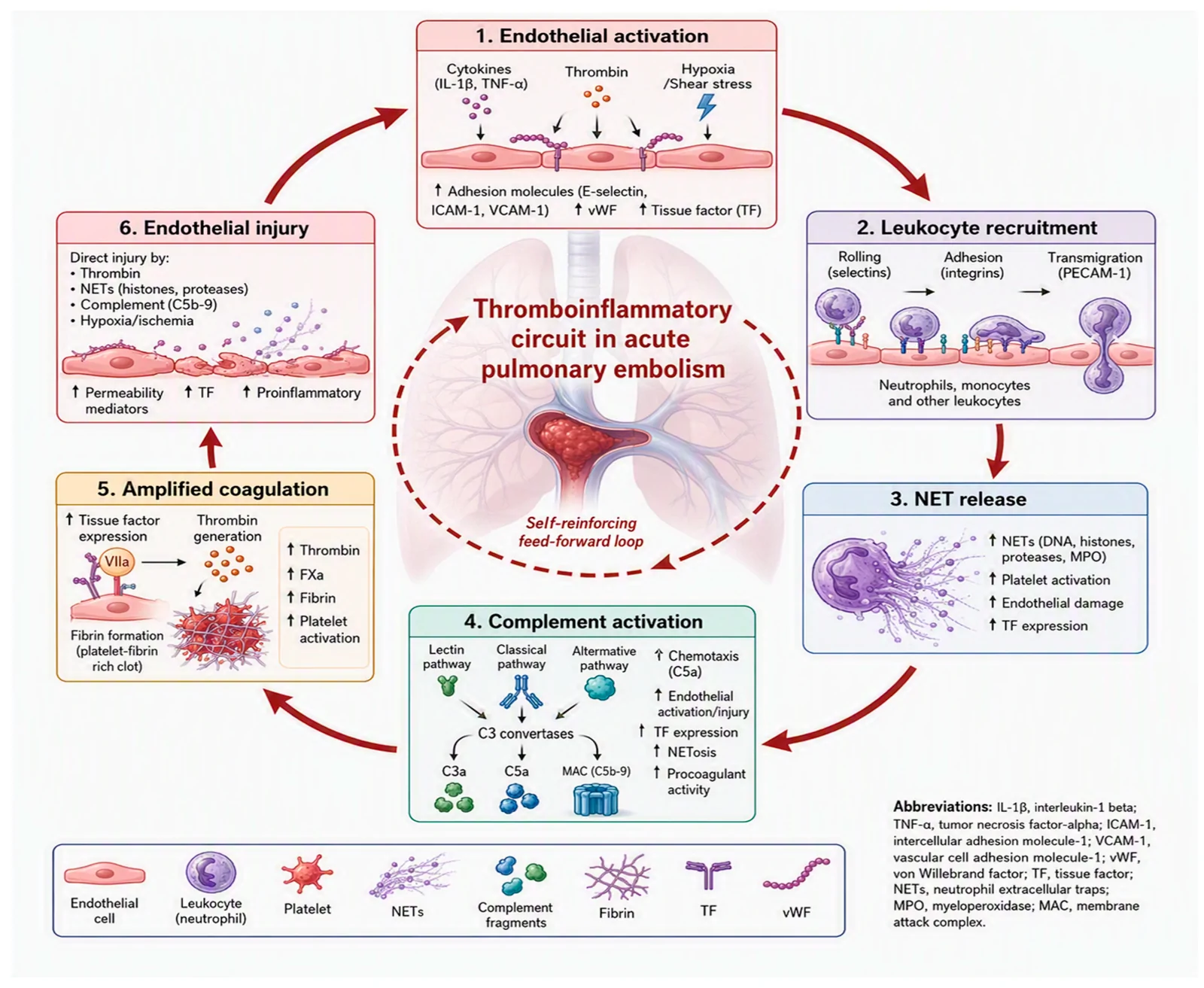
The thromboinflammatory circuit in acute pulmonary embolism. Endothelial activation triggers leukocyte recruitment, neutrophil extracellular trap (NET) release, and complement activation, which in turn amplify coagulation and further endothelial injury, establishing a self-reinforcing feed-forward loop. Note: Figure generated with the assistance of an AI tool (Microsoft Designer). The author reviewed the figure and takes responsibility for its scientific accuracy.

Thromboinflammation is a self-sustaining circuit in which coagulation and innate immunity amplify each other. When a pulmonary artery becomes obstructed, the downstream vascular bed turns ischemic and hypoxic. Within minutes, endothelial cells respond by releasing Weibel-Palade bodies, which expose von Willebrand factor (vWF) and P-selectin on the vessel surface [7]. P-selectin then binds its ligand PSGL-1 on circulating leukocytes, triggering their recruitment to the site of injury (Figure 1, steps 1–2). Once recruited, leukocytes release neutrophil extracellular traps (NETs), which further promote platelet activation and thrombus formation (step 3).

Simultaneously, the complement system is activated, generating anaphylatoxins and membrane attack complexes that intensify the inflammatory response (step 4). This inflammatory milieu drives amplified coagulation: tissue factor expression increases, thrombin generation accelerates, and fibrin formation expands, reinforcing the clot (step 5).

The resulting thrombus causes direct endothelial injury through mechanical stress, complement-mediated damage, and hypoxia-ischemia, which in turn releases more proinflammatory mediators and perpetuates the cycle (step 6). This feed-forward loop explains why thromboinflammation progresses independently of clot size and why outcomes vary so widely among patients [7,8]. Importantly, this cascade represents one face of the same thrombus. It operates in parallel with the myocardial face, driven by right ventricular overload, and the relative dominance of each face in a given patient shapes the trajectory of the disease. Understanding this dual nature is therefore essential for interpreting the biomarker profile of each patient and for moving toward phenotype-guided management.

Neutrophils are the first leukocytes to arrive at sites of thromboinflammation (Figure 1, step 3). Once adherent to the endothelium, they degranulate and release pro-inflammatory mediators, including IL-1β, IL-8, and TNF-α, which help recruit more leukocytes to the area [8].

Neutrophils also deploy neutrophil extracellular traps, or NETs, delicate web-like structures built from decondensed chromatin and studded with histones, elastase, myeloperoxidase, and cathepsin G. First described as a weapon against microbes, NETs are now recognized as powerful prothrombotic players. They promote coagulation through several routes: the DNA scaffold binds vWF and fibrin and supports factor XII activation; histones directly activate platelets via TLR2 and TLR4; elastase degrades tissue factor pathway inhibitor (TFPI); and cathepsin G, together with IL-1α, drives tissue factor (TF) expression on endothelial cells [8,9]. This tendency of innate immunity to actively fuel coagulation is known as immunothrombosis [10].

The complement system adds yet another layer to this cascade, and its role in pulmonary embolism is attracting growing attention (Figure 1, step 4). Clinical observations support this link: in normotensive patients with acute PE, complement consumption and the appearance of activation products have been associated with right ventricular dysfunction and markers of a prothrombotic state [11]. In parallel, high levels of citrullinated histone H3 (citH3), a specific marker of NET formation, have been shown to predict early mortality even among patients previously considered low-risk [12].

At the mechanistic level, the anaphylatoxin C5a stands out as a key mediator. Binding to its receptor C5aR1 (CD88), C5a sets off a series of prothrombotic events: it induces TF expression on monocytes and neutrophils, stimulates further NET release, activates platelets, and impairs fibrinolysis, together making the thrombus harder to dissolve. C5a also increases endothelial permeability, compounding vascular injury (Figure 1, steps 4-5). Notably, blocking C5a or its receptor with a selective C5aR1 inhibitor has been shown to reduce thrombus area and weight and to decrease neutrophil extracellular trap formation in experimental models of vascular thrombosis [13].

Still, direct interventional evidence in PE is lacking, and although a causal role for complement is biologically plausible, it has not yet been proven. Together, these pathways form a self-reinforcing loop that sustains thromboinflammation and ultimately shapes clinical outcomes (Figure 1). This observation helps explain why individuals presenting with a pronounced thromboinflammatory phenotype may benefit from therapeutic strategies that extend beyond conventional anticoagulation [6,10].

### 3.2. Endothelial Dysfunction

The pulmonary endothelium is not a third phenotype but the central effector of the thromboinflammatory face. It regulates vascular tone, permeability, thrombosis resistance, and leukocyte trafficking [7]. In acute PE, all four are disturbed, and the degree of endothelial injury closely mirrors clinical severity. Loss of nitric oxide (NO) bioavailability occurs early. Endothelial nitric oxide synthase (eNOS) normally generates NO continuously, sustaining vasodilation and limiting platelet activation through soluble guanylate cyclase (sGC) and cyclic guanosine monophosphate (cGMP).

During PE, ischemia and reperfusion generate superoxide, which reacts rapidly with NO to form peroxynitrite [14]. Peroxynitrite oxidizes tetrahydrobiopterin (BH4) and uncouples the synthase, so the enzyme itself begins to produce superoxide instead of NO [14]. This is not merely a shortage of NO; it evolves into a self-perpetuating cycle in which pulmonary vasoconstriction, enhanced platelet reactivity, and increased leukocyte transmigration continuously amplify one another [14,15].

Two circulating markers provide indirect access to this pathway: asymmetric dimethylarginine (ADMA), an endogenous inhibitor of eNOS, and endothelin-1 (ET-1), a potent vasoconstrictor. Both rise in acute PE and correlate with severity [15,16].

At the same time, the endothelial surface shifts toward a procoagulant phenotype. Nuclear factor kappa B (NF-κB)-dependent upregulation of intercellular adhesion molecule-1 (ICAM-1) and vascular cell adhesion molecule-1 (VCAM-1) promotes firm leukocyte adhesion and transmigration [7]. TF is induced, while thrombomodulin and endothelial protein C receptor (EPCR) are lost, reducing activated protein C (APC) generation precisely where it would be most useful [8]. Structural integrity also fails: histamine, bradykinin, and vascular endothelial growth factor (VEGF) destabilize vascular endothelial cadherin (VE-cadherin) at adherens junctions, increasing paracellular leakage and worsening gas exchange [7].

Two cellular markers extend this picture. Endothelial microparticles (EMPs) are shed during activation and apoptosis; they rise acutely and expose phosphatidylserine and TF, so they act as both effectors and reporters. By contrast, circulating cluster of differentiation 34-positive (CD34+) progenitor cells reflect reparative capacity. In a follow-up study after PE, lower counts were associated with residual pulmonary vascular obstruction, suggesting that impaired vascular repair may contribute to incomplete recovery [17].

However, neither marker is ready for routine use, largely because flow cytometric definitions differ across laboratories and the results are not yet comparable between centers. Together, these mechanisms and markers illustrate how endothelial dysfunction amplifies the thromboinflammatory face, creating a vascular environment that sustains coagulation, inflammation, and impaired perfusion long after the initial obstruction.

### 3.3. Right Ventricular Dysfunction

The consequences of endothelial injury and thromboinflammation extend beyond the vascular lumen to the myocardium itself. Death in acute PE is usually the consequence of right ventricular failure. The right ventricle is a thin-walled chamber adapted to a low-resistance circulation; it tolerates volume loading relatively well but decompensates rapidly when faced with sudden pressure overload [18,19]. When a substantial portion of the pulmonary circulation is abruptly obstructed, and when hypoxic vasoconstriction plus serotonin and thromboxane release further raise resistance, afterload can exceed the ventricle’s capacity.

The resulting sequence is well defined. Pressure overload dilates the right ventricle, and by the Law of Laplace, dilation increases wall stress and myocardial oxygen demand at the very moment when supply is already compromised [18]. Tricuspid annular dilation produces functional regurgitation, reducing forward flow and raising right atrial pressure [18].

Because the two ventricles share the septum and the pericardial space, right ventricular dilation shifts the septum leftward, limits left ventricular filling, lowers stroke volume, and contributes to systemic hypotension [18]. Falling aortic pressure then reduces right coronary perfusion and further depresses contractility, closing a vicious cycle that can be difficult to interrupt once established [19].

Neurohormonal responses do not compensate effectively and may worsen the problem. Activation of the renin-angiotensin-aldosterone system (RAAS) expands intravascular volume and distends an already failing ventricle, while adrenergic stimulation increases heart rate and oxygen demand in an underperfused myocardium [18,19]. Natriuretic peptide release is proportional to wall stress, which is why it is clinically useful, even though it does not itself reverse hemodynamic deterioration.

**Figure 2:**
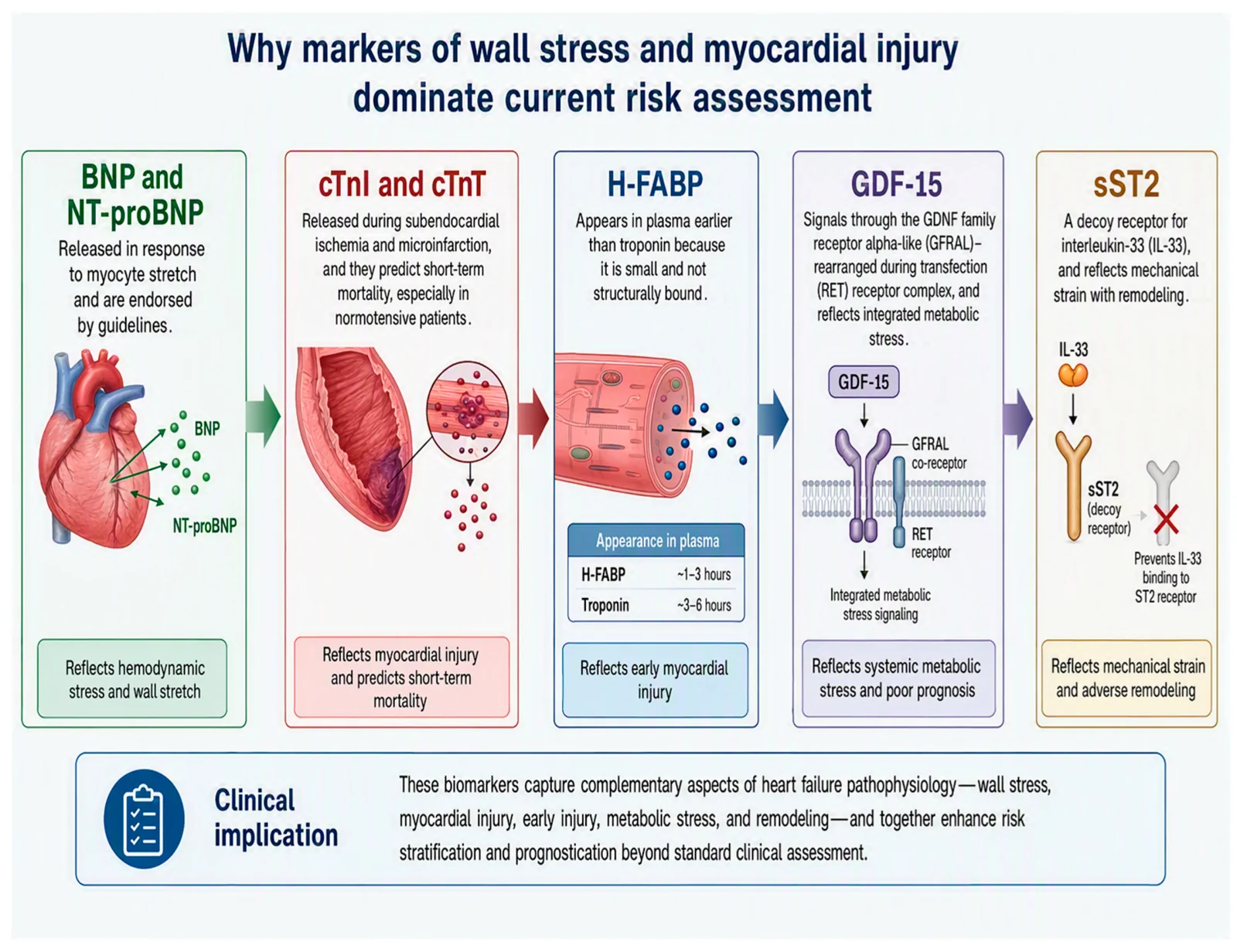
Wall stress and myocardial injury biomarkers reflect distinct pathophysiological processes in acute PE. BNP and NT-proBNP [20]: released in response to ventricular wall stretch; indicate hemodynamic overload. cTnI and cTnT [21]: cardiac troponins released upon myocardial injury; predictors of infarction and ventricular dysfunction. H-FABP [22]: heart fatty acid binding protein; rises early after myocardial ischemia. GDF-15: a TGF-β superfamily cytokine; reflects inflammatory and apoptotic stress. sST2: soluble receptor for interleukin-33; a marker of fibrotic remodeling and mechanical stress [23,24]. Note: Figure generated with the assistance of an AI tool (Microsoft Designer). The author reviewed the figure and takes responsibility for its scientific accuracy.

This sequence explains why markers of wall stress and myocardial injury dominate current risk assessment. BNP and NT-proBNP are released in response to myocyte stretch and are endorsed by guidelines [20]. Cardiac troponins I and T (cTnI and cTnT) are released during subendocardial ischemia and microinfarction, and they predict short-term mortality, especially in normotensive patients [21]. Heart-type fatty acid binding protein (H-FABP) appears in plasma earlier than troponin because it is small and not structurally bound [22]. Growth differentiation factor 15 (GDF-15), which signals through the GDNF family receptor alpha-like (GFRAL) and rearranged during transfection (RET) receptor complex, and soluble suppression of tumorigenicity 2 (sST2), a decoy receptor for interleukin-33 (IL-33), reflect integrated metabolic stress and mechanical strain with remodeling, respectively, although the additional value of either marker over established tests remains under study [23,24] (Figure 2).

Together, these mechanisms and markers illustrate the myocardial face of the thrombus. The mechanical cascade from pressure overload to ventricular interdependence, the neurohormonal responses that fail to compensate, and the biomarkers that reveal wall stress and subendocardial injury all converge on a single point: the thrombus does not merely obstruct flow; it reprograms the right ventricle into a state of metabolic and mechanical distress. This myocardial face complements the thromboinflammatory face described in the previous section, and together they explain why pulmonary embolism is not a single event but a sustained pathophysiological process that extends far beyond the initial obstruction.

## 4. Established Biomarkers

### 4.1. D-Dimer

D-dimer is a fibrin degradation product generated when plasmin cleaves cross-linked fibrin. It therefore reflects the balance between coagulation and fibrinolysis, but it does not indicate where a thrombus is located. Its main clinical value lies in exclusion algorithms, especially when combined with the Wells rule or the revised Geneva score, because its sensitivity is very high [25,26]. Its weakness is poor specificity: many conditions increase fibrin turnover, including infection, malignancy, pregnancy, trauma, and the postoperative state [25]. To improve diagnostic accuracy, particularly in older patients, age-adjusted thresholds, calculated as age × 10 μg/L for patients over 50, have been proposed and evaluated in both prospective and retrospective cohorts with consistent results [27,28,29].

Beyond diagnosis, D-dimer levels also correlate with embolic burden and short-term mortality: higher levels tend to reflect larger proximal clots and worse outcomes [28]. However, because of its low specificity, D-dimer alone cannot be used for risk stratification, highlighting the need for additional prognostic tools.

### 4.2. Cardiac Troponins

Cardiac troponins I and T (cTnI and cTnT) are components of the thin filament regulatory complex and enter the circulation when cardiomyocyte membrane integrity is lost. In acute PE, elevation reflects the subendocardial ischemia and microinfarction caused by the supply-demand mismatch described earlier [21]. Troponin positivity is associated with a several-fold increase in short-term mortality [30].

Troponin is also a key component of the Bova score, which stratifies normotensive patients into distinct risk categories, and it is used in European guidelines to differentiate between intermediate-low and intermediate-high risk groups [20,31]. However, this marker lacks specificity for pulmonary embolism and can be elevated in various other conditions, including acute coronary syndromes, myocarditis, sepsis, and renal dysfunction. The implementation of high-sensitivity cardiac troponin assays increases the detection of positive cases, although this benefit comes at the cost of reduced specificity. Recent meta-analysis data confirm the prognostic value of troponin, while also highlighting that its clinical performance depends heavily on both the specific assay and the chosen threshold [32].

### 4.3. Natriuretic Peptides

B-type natriuretic peptide (BNP) and N-terminal pro-B-type natriuretic peptide (NT-proBNP) are released in response to myocardial stretch. NT-proBNP is often preferred analytically because of its longer half-life. Pooled analyses show that elevated natriuretic peptide concentrations are associated with right ventricular dysfunction, complicated in-hospital course, and 30-day mortality [33].

When used together, natriuretic peptides and troponin offer a more complete view of cardiac status in normotensive patients with acute PE. The reason lies in their biological difference: troponin is released when myocardial cells are injured, whereas natriuretic peptides rise in response to ventricular wall stress. Because each marker reflects a distinct pathophysiological process, combining them improves discrimination beyond what either could achieve alone [34]. This dual-marker strategy, however, is not without practical limitations. Assay standardization across platforms remains suboptimal, and clinical variables such as renal dysfunction, obesity, and pre-existing heart failure can independently alter peptide concentrations. As a result, fixed numerical thresholds may not translate reliably across different patient populations [35].

Nevertheless, the negative predictive value of these markers remains high enough to support outpatient management in carefully selected low-risk patients [20]. A comprehensive summary of all biomarkers discussed in this review, including their associated molecular phenotype, mechanism of release, current clinical utility, and principal limitations, is provided in Table 1A,B.

## 5. Emerging Biomarkers

### 5.1. Growth Differentiation Factor 15

Growth differentiation factor 15 (GDF-15) belongs to the transforming growth factor beta superfamily and is expressed at low levels across most tissues. However, its expression rises sharply under conditions of oxidative stress, hypoxia, mechanical load, and inflammatory signaling through IL-1β and TNF-α [23]. These triggers are particularly relevant in cardiomyocytes, macrophages, and endothelial cells. Once released, GDF-15 exerts its central effects through the brainstem GFRAL-RET receptor complex, which mediates both cardiovascular protection and the reduction in hypertrophy and apoptosis [23].

Because a single biomarker integrates inflammatory, hypoxic, and mechanical stress, GDF-15 is biologically well suited to a disease in which all three processes overlap. This theoretical advantage is borne out by strong clinical evidence. The key clinical dataset comes from a prospective cohort of 123 consecutive patients with confirmed acute PE. In this study, 101 patients (82%) had admission concentrations above the upper reference limit of 1200 ng/L [36]. Values were substantially higher in patients who developed complications within 30 days, and the difference was highly significant [36].

On multivariable analysis, the marker remained an independent predictor of a complicated 30-day course after adjustment for clinical variables, cTnT, natriuretic peptide, and echocardiographic findings [36]. Its discriminatory performance also exceeded that of established markers, and it predicted long-term mortality as well [36]. A later study confirmed its independent prognostic value, showing that concentrations correlate with inflammatory activation and recurrent venous thromboembolism (VTE) [37].

Despite these promising findings, two limitations still constrain translation into routine practice. No assay is standardized across platforms, and concentrations rise with age, renal impairment, malignancy, and heart failure [38]. These factors make it unlikely that a threshold derived in one population will transfer cleanly to another.

### 5.2. Heart-Type Fatty Acid Binding Protein

Heart-type fatty acid binding protein (H-FABP) is a small cytosolic protein that transports fatty acids within the cell. Its most relevant feature is its size: troponin remains bound in the cell structure, whereas H-FABP is small and freely soluble, allowing it to enter the bloodstream rapidly after myocardial injury. As a result, the marker reaches peak levels within about two hours, which makes it attractive for early risk assessment in acute settings [22].

This theoretical advantage has been confirmed in clinical studies. In a cohort of 107 consecutive patients with confirmed PE, 29 (27%) had H-FABP levels above 6 ng/mL at admission. Among them, 12 (41%) developed an adverse clinical course, whereas all patients with normal levels had an uneventful 30-day outcome [22]. On multivariable analysis, H-FABP independently predicted PE-related death or major complications, while troponin and natriuretic peptide did not [22]. Combining H-FABP with echocardiography proved particularly informative: a positive test carried a complication rate of 23.1% even in the presence of a normal echocardiogram, which increased to 57.1% when right ventricular dysfunction was also present [22].

The strongest evidence comes from patients with the highest degree of prognostic uncertainty, namely those with normal blood pressure but echocardiographic signs of right ventricular overload. In this group, 10 of 14 patients (71%) with a positive test deteriorated and required inotropic support, and 8 died. In contrast, none of the 87 patients with a negative test worsened, required inotropes, or died in hospital [49]. A dedicated H-FABP assay and a fast prognostic score for normotensive PE have been developed, and comparative studies support the superiority of H-FABP over troponin and myoglobin for rapid stratification [50,51]; independent cohorts and meta-analyses confirm its additional prognostic value in normotensive disease [49,52]. The marker has also shown prognostic value in acute PE [52], appears largely independent of symptom duration [53], and has been studied in chronic thromboembolic pulmonary hypertension [54].

In spite of this consistent evidence, H-FABP is still not widely used in clinical practice. Assays are not universally available, and circulating levels are influenced by renal clearance, as well as by conditions such as acute coronary syndromes, heart failure, and skeletal muscle injury [51,52]. These factors hinder interpretation and limit its routine adoption.

### 5.3. Soluble Suppression of Tumorigenicity 2

Soluble suppression of tumorigenicity 2 (sST2) is the circulating, soluble form of the interleukin-1 receptor family member ST2. It acts as a decoy receptor that binds interleukin-33 (IL-33) and prevents its engagement with the membrane-bound receptor ST2L, thereby blocking a signaling axis that protects the myocardium from hypertrophy, fibrosis, and apoptosis under biomechanical strain [24]. Cardiomyocytes, cardiac fibroblasts, and endothelial cells release sST2 in response to mechanical stretch and inflammatory injury, which is why circulating concentrations reflect the integrated burden of right ventricular pressure overload and maladaptive remodeling in acute PE [24].

The clinical evidence in PE remains at an early stage. A recent pilot study focusing on intermediate-risk subgroups reported that sST2 discriminated intermediate-high from intermediate-low risk patients and was associated with short-term adverse events, adding modest incremental value over established markers [24]. These findings are biologically coherent with the role of sST2 as a marker of strain and remodeling, but they derive from small, single-center cohorts without pre-specified thresholds or external validation. Until larger multicenter studies confirm its incremental value over troponin and natriuretic peptides, sST2 should be regarded as a promising candidate for multi-marker panels rather than a standalone clinical tool.

### 5.4. Complement Activation Products

Complement is not only a host-defense system but also a driver of thromboinflammation, and its activation products have emerged as candidate biomarkers in acute PE (see Section 3.1). In normotensive patients, complement consumption and the appearance of activation products have been associated with right ventricular dysfunction and a prothrombotic state [11]. Citrullinated histone H3 (citH3), a specific marker of NET formation, predicts early mortality even among patients previously classified as low-risk [12]. These observations are mechanistically supported by experimental data showing that the anaphylatoxin C5a promotes tissue factor expression, platelet activation, and impaired fibrinolysis [13].

Despite their biological appeal, complement-derived markers face practical obstacles. Assays for individual activation products are not harmonized across laboratories, reference ranges are poorly defined, and most clinical data come from single-center observational cohorts [11,12]. As with sST2, these markers are best viewed as research tools that could enrich future phenotype-directed panels rather than as tests ready for routine use.

### 5.5. Inflammatory Indices from the Routine Blood Count

A routine blood count carries relevant information about systemic inflammation. Since platelets, neutrophils, monocytes, and lymphocytes take part in the same thromboinflammatory pathway, combining these cell populations into simple ratios has proven to be an inexpensive and practical way to estimate the inflammatory state. The systemic immune-inflammation index (SII), for instance, is calculated as platelets × neutrophils/lymphocytes [41]. The systemic inflammatory response index (SIRI), in turn, uses neutrophils × monocytes/lymphocytes [42]. A more comprehensive version, the aggregate index of systemic inflammation (AISI), incorporates the three cell populations of the innate response in the numerator and divides the product by lymphocytes: neutrophils × platelets × monocytes/lymphocytes [43].

Importantly, these newer indices are built upon two older and well-established metrics, the neutrophil-to-lymphocyte ratio (NLR) and the platelet-to-lymphocyte ratio (PLR), which have been widely validated in cardiovascular and oncological research [41,42,43]. In that sense, SII, SIRI, and AISI can be understood not as new concepts, but as refinements of a familiar idea, now extended to incorporate a broader view of the immune response.

The biological rationale behind these indices is straightforward. The numerator reflects the pro-inflammatory and pro-thrombotic arm of the immune response, while the denominator represents its regulatory counterpart. By expressing one relative to the other, these indices capture the overall balance of the immune system in a single, bedside-available number. Because the blood count is inexpensive and routinely collected, these ratios can be calculated without any additional cost or delay [39]. In clinical practice, they have been able to discriminate non-massive from severe presentations and to track disease progression with good sensitivity [39].

Nevertheless, interpretation requires caution for three main reasons. First, reference values are not universal: these indices vary with age, sex, and smoking status, so identical numbers may carry different meanings across individuals [40]. Second, most indices were originally developed and validated in oncology, and their performance in that setting does not necessarily transfer to other diseases [41,42]. Third, confounding conditions such as superimposed infection, occult malignancy, or corticosteroid use can alter the cell counts independently of the disease under study. For these reasons, these indices are better regarded as useful clues than as definitive diagnostic tools. Prospective studies with pre-defined thresholds will be required before they can be adopted in routine clinical practice.

### 5.6. MicroRNAs

MicroRNAs (miRNAs) are short, non-coding RNA molecules of 20 to 24 nucleotides that regulate gene expression by binding to target messenger RNAs [44]. Beyond their intracellular role, they circulate in the bloodstream, protected from degradation by exosomes or protein complexes such as Argonaute 2 and high-density lipoprotein [48]. This structural stability allows them to remain intact in plasma for extended periods, an advantage over more fragile protein-based biomarkers [44,48].

In venous thromboembolism (VTE), systematic reviews show moderate diagnostic performance for circulating miRNAs, though with substantial heterogeneity that reflects the early stage of the field [45]. Among specific candidates, microRNA-1233 (miR-1233) stands out for its relative specificity for acute pulmonary embolism [46], and it has been evaluated in panels designed to distinguish PE from acute myocardial infarction [47]. The rationale for panels rather than single miRNAs is that thrombosis involves multiple pathways, so a combination of miRNAs mapping to different biological processes captures the disease process more completely than any single molecule.

The concept is appealing, but practical barriers currently prevent routine clinical use. Quantification requires technically demanding methods such as reverse transcription quantitative PCR or sequencing, no consensus normalizer exists across laboratories, and hemolysis is a critical confounder. Even minor red blood cell rupture can contaminate plasma with platelet- and erythrocyte-derived miRNAs, altering the measured profile [48]. These methodological problems are amplified by the fact that most published cohorts are small and single-center, limiting the generalizability of the findings [48].

Beyond these practical challenges, however, circulating miRNAs remain biologically significant, because many individual species map directly onto the thromboinflammatory pathways driving VTE [44]. They should not be dismissed as premature, but rather recognized as a maturing biomarker class. The next steps will require large, multicenter prospective studies, standardized pre-analytical protocols, and validated normalizers. Until these requirements are met, miRNAs are best regarded as a rich source of biological insight and a promising direction for future biomarker development, rather than tools ready for immediate clinical implementation.

## 6. Kinetics and Serial Measurement

**Figure 3:**
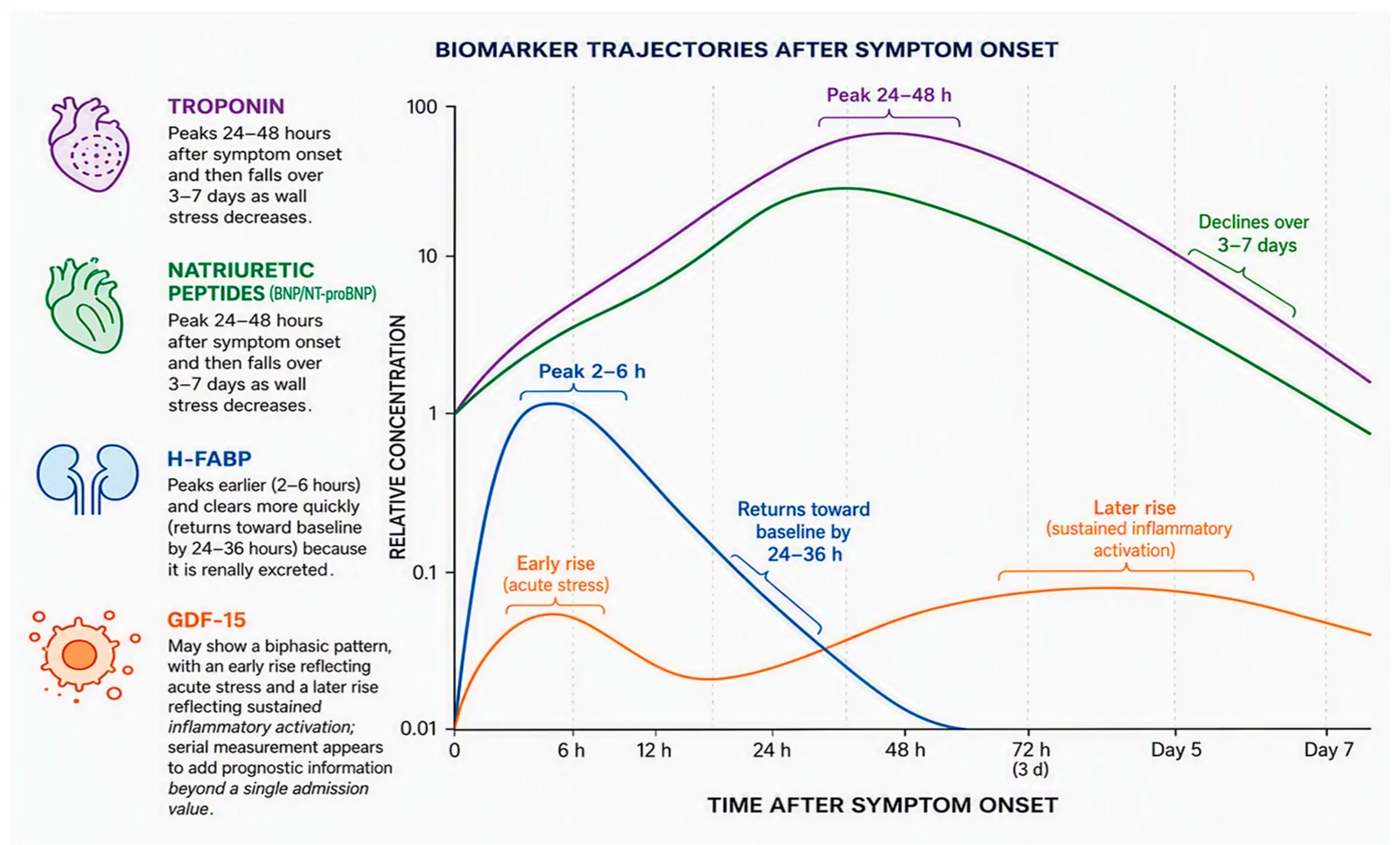
Schematic release kinetics of circulating biomarkers over the first 72 h after symptom onset in acute pulmonary embolism. H-FABP [22] rises earlies(peak 2–6 h) and clears quickly; cardiac troponin and natriuretic peptides peak between 24 and 48 h and decline gradually over 3–7 days [55,57]; GDF-15 shows a biphasic pattern, with an early rise reflecting acute stress followed by sustained inflammatory activation [36]. Note: Figure generated with the assistance of an AI tool (Microsoft Designer). The author reviewed the figure and takes responsibility for its scientific accuracy.

A single measurement describes severity at one moment. Repeated measurements describe trajectory, which is closer to the question clinicians actually need to answer: will a currently stable patient deteriorate? Release kinetics follow the underlying biology. Troponin and natriuretic peptides generally peak 24 to 48 h after symptom onset and then fall over three to seven days as wall stress decreases [55]. H-FABP peaks earlier and clears more quickly because it is renally excreted [22], a feature that, together with its independence from symptom duration [53], supports its use across variable presentation times. GDF-15 may show a biphasic pattern, with an early rise reflecting acute stress and a later rise reflecting sustained inflammatory activation; serial measurement appears to add prognostic information beyond a single admission value [36] (Figure 3).

Direct evidence remains limited. In a proof-of-concept study, natriuretic peptide, troponin, uric acid, and lactate were measured serially over 72 h in patients with intermediate-high-risk disease [56]. Natriuretic peptide and troponin peaked at 24 h and were associated with hemodynamic deterioration and vasopressor requirement, while uric acid and lactate remained elevated in patients who deteriorated, consistent with ongoing tissue hypoxia and impaired oxygen delivery [56]. A rising trajectory carried a much higher risk of adverse outcome than a stable or falling one [56].

Because the cohort was small and single-center, the estimate is imprecise and should be treated as hypothesis-generating rather than practice-changing. If larger studies confirm these findings, serial sampling could help identify patients who need earlier escalation, guide the timing of catheter-directed or systemic reperfusion, provide biochemical confirmation that reperfusion has been successful, and identify those safe for earlier discharge. The price of this approach is repeated phlebotomy, standardized sampling, and additional assay cost, which would have to be justified by fewer complications or shorter hospital stays.

## 7. Integration of Biomarkers into Clinical Algorithms

In patients with suspected acute PE, biomarkers can complement clinical decision rules and reduce unnecessary imaging. Diagnostically, in patients with low or intermediate pretest probability, a negative age-adjusted D-dimer excludes the diagnosis without imaging, whereas a positive result leads to CTPA. In patients with high pretest probability, D-dimer has no role and imaging should be obtained directly. Prognostically, troponin and natriuretic peptides are measured only after the diagnosis is established, where they inform management rather than diagnosis.

**Figure 4:**
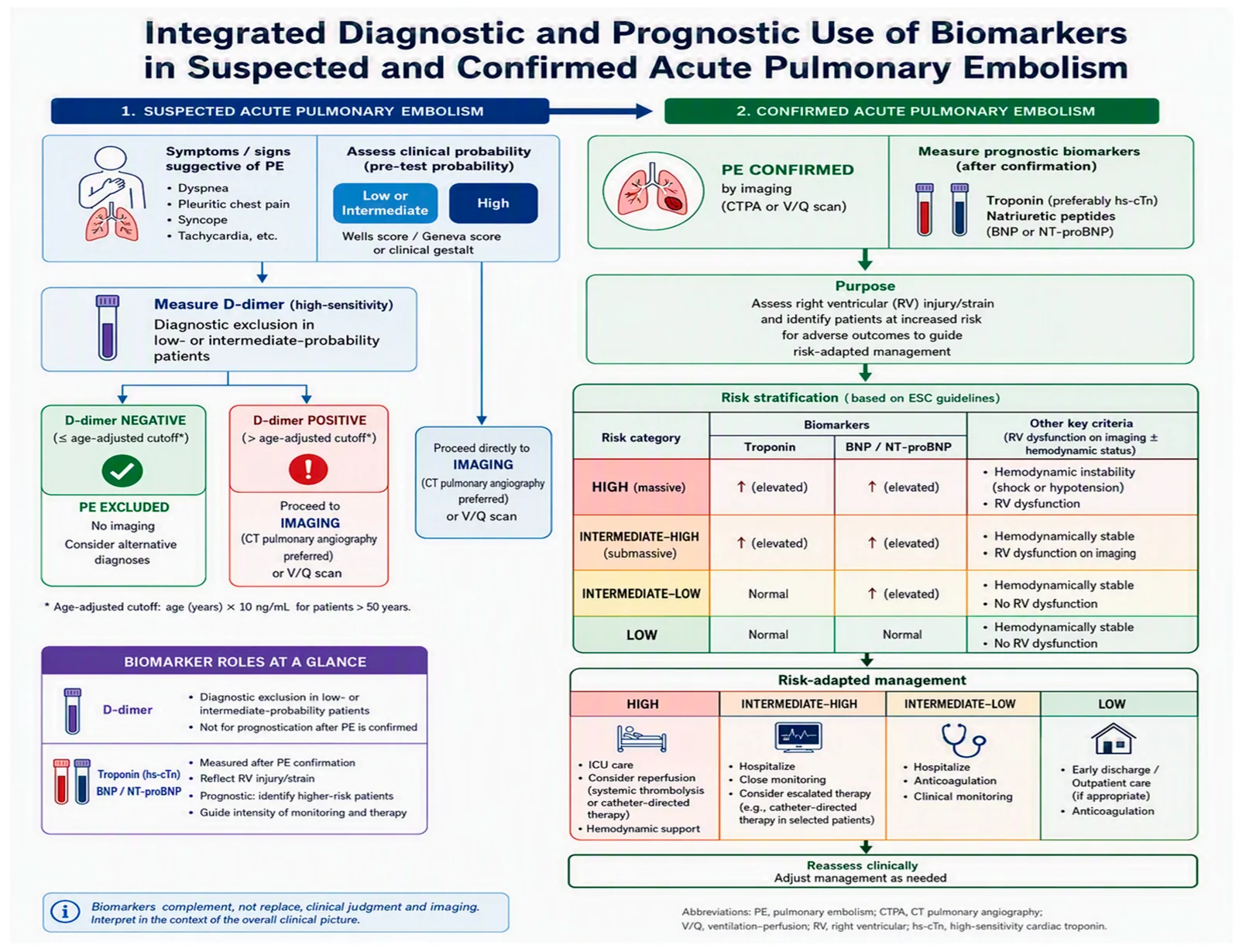
Integrated diagnostic and prognostic use of biomarkers in suspected and confirmed acute pulmonary embolism. D-dimer is used for diagnostic exclusion in low- or intermediate-probability patients; troponin and natriuretic peptides are measured after confirmation to guide risk-adapted management. Note: Figure generated with the assistance of an AI tool (Microsoft Designer). The author reviewed the figure and takes responsibility for its scientific accuracy.

Once PE is confirmed, biomarkers are interpreted together with hemodynamic status and right ventricular imaging to define risk and guide treatment, in line with guideline-based frameworks [20,25] (Figure 4).

Hypotension or shock defines high-risk disease regardless of biomarker values. GDF-15, H-FABP, sST2, and routine blood-count indices are not included in this algorithm because none is currently endorsed by guidelines [20,25].

**Figure 5:**
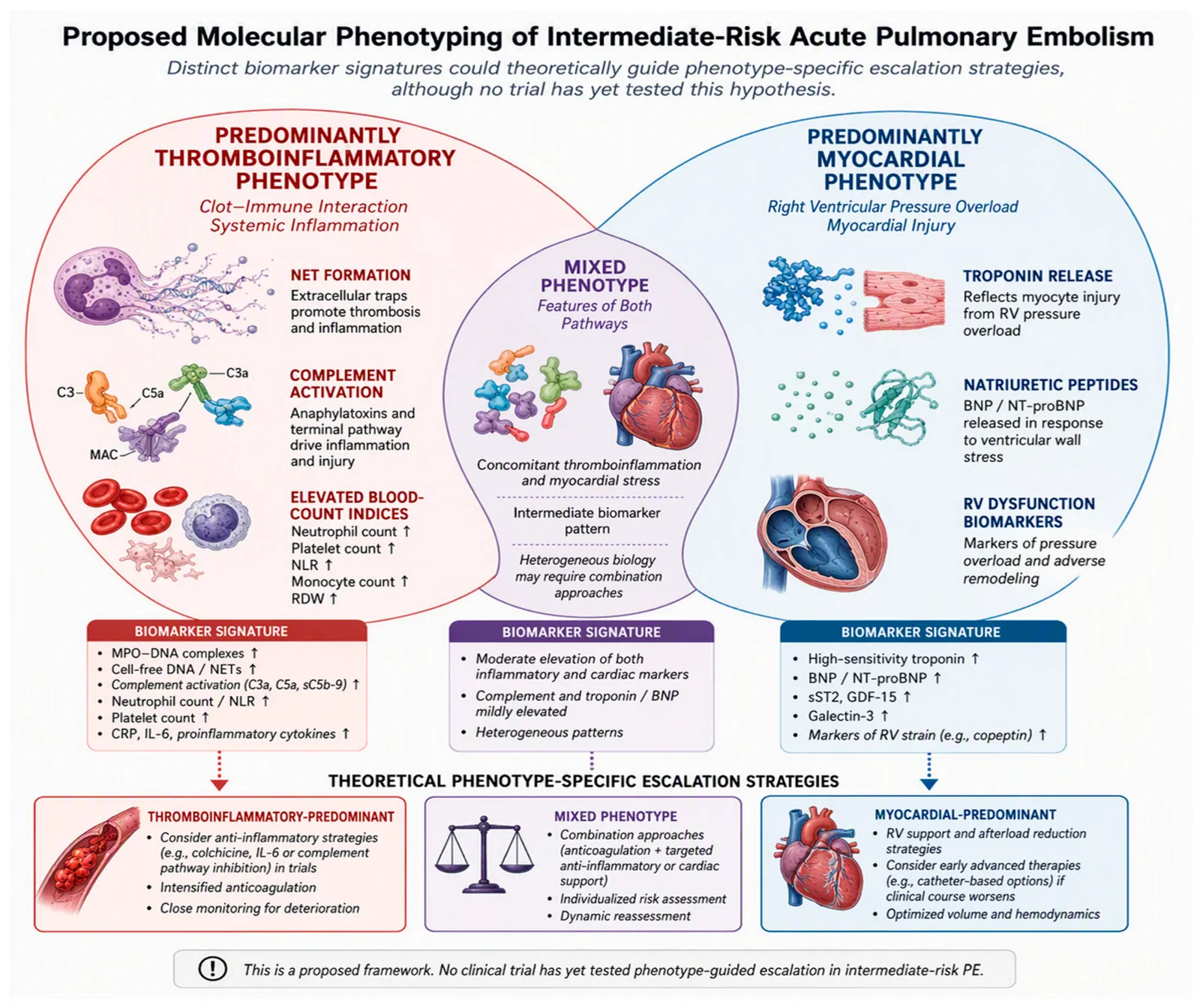
Hypothetical model of molecular phenotyping in intermediate-risk acute pulmonary embolism. This schematic illustrates a conceptual proposal that has not been validated in clinical trials. The predominantly thromboinflammatory phenotype (left) is characterized by NET formation, complement activation, and elevated blood-count indices, whereas the predominantly myocardial phenotype (right) is characterized by troponin and natriuretic peptide release reflecting pressure-overload injury. The overlapping central region represents mixed phenotypes. Whether distinct biomarker signatures could guide phenotype-specific escalation strategies remains to be tested in prospective studies. Note: Figure generated with the assistance of an AI tool (Microsoft Designer). The author reviewed the figure and takes responsibility for its scientific accuracy.

The longer-term goal is not simply to add more markers to existing scores, but to identify molecular phenotypes. Intermediate-risk disease is the obvious target because it is common, biologically heterogeneous, and the setting in which escalation decisions are genuinely uncertain. One can imagine separating a predominantly thromboinflammatory phenotype, marked by a high burden of NETs, complement consumption, and elevated blood-count indices, from a predominantly myocardial phenotype, marked by troponin and natriuretic peptide release, and then testing different treatments for each group [6,10,11] (Figure 5).

Multi-marker panels analyzed with methods that can handle trajectories, together with proteomic and transcriptomic profiling, may eventually make this possible [4,5]. For now, however, this remains a research proposal. No trial has yet randomized patients on the basis of a biomarker-defined phenotype.

Anticoagulation remains the cornerstone of treatment [20,25], and its benefit lies in preventing clot propagation and recurrent embolism rather than in directly reversing established right ventricular failure or the thromboinflammatory response already in progress [25].

This distinction matters for biomarker-guided therapy: a patient whose trajectory is deteriorating despite adequate anticoagulation is precisely the patient in whom escalation, catheter-directed or systemic reperfusion, or hemodynamic support, should be considered, and serial biomarkers could identify this trajectory earlier than clinical deterioration alone. Equally, a falling or stable biomarker profile in a low-risk patient could support earlier discharge. Conversely, markers that remain elevated after reperfusion may indicate that the inflammatory cascade has not been interrupted, prompting a search for residual obstruction or a second process. None of these uses is yet evidence-based, but together they define a clear research agenda.

## 8. Discussion

### 8.1. Do Molecular Patterns Track Prognosis?

The central question raised by the two-phenotype framework is whether the molecular signature of thromboinflammation truly tracks the risk of early death and clinical deterioration, or whether it merely reflects disease severity in a generic way. The available evidence favors the first reading. In the prospective multicenter COPE cohort of 5036 hemodynamically stable patients with acute PE, the TELOS score, which adds hyperlactatemia to troponin, right ventricular dysfunction, blood pressure, and heart rate, showed the best discrimination for in-hospital death or clinical deterioration (c-statistic 0.722) and for PE-related death (c-statistic 0.780) among the models compared [57]. This result is mechanistically informative because circulating lactate is more than a generic marker of hypoperfusion: in acute PE it has been linked to prothrombotic fibrin clot properties and to enhanced formation of neutrophil extracellular traps, two features of the thromboinflammatory circuit described in Section 3.1. The same pattern appears in direct molecular readouts. Complement activation products and citrullinated histone H3, which report the same immunothrombotic pathways, have been associated with right ventricular dysfunction and with early mortality even among patients classified as low risk by conventional scores [11,12]. Taken together, these findings suggest that a worse short-term outcome is not biologically silent. It tends to be accompanied by a recognizable molecular pattern in which NETosis, complement consumption, endothelial injury, and impaired fibrinolysis coexist. In short, the thromboinflammatory face of the thrombus appears to be both a driver and a measurable predictor of clinical deterioration. This convergence between clinical scores and direct molecular readouts supports the idea that prognosis in acute PE has a molecular substrate, and that the intermediate-risk group is where this substrate most needs to be separated into distinct phenotypes.

### 8.2. Thromboinflammation as a Therapeutic Target: Lessons from COVID-19

If coagulation and innate immunity reinforce each other and worsen outcomes, interrupting this circuit may offer therapeutic opportunities beyond anticoagulation. Anticoagulation mainly prevents thrombus propagation and recurrence, but it does not extinguish a thromboinflammatory cascade that is already underway. The COVID-19 pandemic provided a large-scale clinical lesson here. Severe SARS-CoV-2 infection behaved as a thromboinflammatory syndrome, with microthrombosis, NET formation, complement activation, and endothelial injury progressing despite standard thromboprophylaxis, and with adjunctive immunomodulation, most notably IL-6 pathway blockade, becoming part of accepted management [58]. The same logic applies to acute PE. In animal models, neutrophil depletion or blockade of C5a/C5aR1 reduces thrombus burden and right ventricular dysfunction, and the damage-associated mediator HMGB1 has emerged as an upstream orchestrator that links NET recruitment, platelet activation, and vascular remodeling. Anti-HMGB1 has therefore been proposed as a way to prevent residual pulmonary hypertension and post-thrombotic sequelae [8,13,58]. The immunomodulatory hypothesis does not displace anticoagulation; it refines it by pointing to phenotype-directed anti-inflammatory or network-targeting interventions for patients whose course is dominated by the thromboinflammatory face. Clinical translation will require markers that identify this face reliably and prospectively, which is precisely the phenotype-guided approach advocated throughout this review. For now, the evidence remains preclinical or observational, and no trial has yet selected PE patients for immunomodulatory therapy on the basis of a molecular phenotype. This gap does not discourage the hypothesis; it defines the next generation of studies.

Taken together, these two lines of evidence converge on a single proposition: the thromboinflammatory substrate that worsens short-term prognosis is the same substrate that phenotype-directed immunomodulation would need to interrupt [57,58]. The intermediate-risk group is where both questions must be answered together.

## 9. Challenges and Unmet Needs

Several barriers still separate the biomarkers discussed here from routine clinical use. Most emerging markers lack commercially available assays that are harmonized across platforms and traceable to a reference material, so thresholds cannot be transferred reliably from one setting to another. Most studies are single-center cohorts, and prospective multicenter validation with pre-specified thresholds has not yet been done. No trial has shown that acting on a biomarker result improves outcome, which is the type of evidence required for guideline endorsement; this is why current recommendations remain limited to troponin and natriuretic peptides [20,25].

Comorbidity, especially renal impairment, heart failure, malignancy, and infection, also shifts concentrations in ways that a single measurement cannot resolve. Finally, the added cost of multi-marker testing must be justified against a clinical benefit that remains unproven. Recent reviews echo this conclusion and single out intermediate-risk disease as the key setting for future prospective work [4,5,6].

## 10. Conclusions

Acute pulmonary embolism is best understood as a disease with two overlapping phenotypes. One is driven by inflammation amplifying coagulation; the other by the right ventricle straining under sudden pressure overload. Both operate simultaneously in most patients, and both leave traces in the blood. Troponin and natriuretic peptides reflect cardiac strain, D-dimer the thrombotic burden, and markers such as GDF-15 bridge the two.

This view helps explain why some patients deteriorate. A poor early outcome is accompanied by a measurable signature of intense inflammation, impaired clot breakdown, and blood vessel injury, information that adds to what clinical scores already capture. The same signature indicates where treatment may go beyond anticoagulation. The COVID-19 experience and experimental models suggest that interrupting this inflammatory response could alter the course of pulmonary embolism, a possibility that now requires prospective evaluation.

What makes the disease dangerous and what a future therapy should target are ultimately the same process. The intermediate-risk group, where current assessment is least reliable, offers the right setting for testing this connection. Assay standardization, defined thresholds, and multicenter validation remain necessary before these markers can guide practice, but the path ahead is clear.

## Figures and Tables

**Table 1:** (**A**) Established biomarkers in acute pulmonary embolism endorsed by current guidelines. (**B**) Emerging and investigational biomarkers in acute pulmonary embolism.

(**A**)				
**Biomarker**	**Predominant Phenotype**	**Mechanism of Release**	**Clinical Utility (Validated)**	**Principal Limitations**
D-dimer [25,26,27,28,29]	Thromboinflammatory	Plasmin cleavage of cross-linked fibrin	Diagnostic exclusion in patients with low or intermediate clinical probability; correlates with embolic burden	Low specificity; age-adjusted thresholds required in patients >50 years; not suitable alone for risk stratification
Cardiac troponins (cTnI/cTnT) [20,21,30,31,32]	Myocardial	Release upon loss of myocyte membrane integrity due to subendocardial ischemia	Short-term mortality prediction; key component of the Bova score and ESC intermediate-risk stratification (intermediate-low vs. intermediate-high)	Not PE-specific; elevated in ACS, myocarditis, sepsis, renal dysfunction; assay-dependent thresholds
BNP/NT-proBNP [20,30,31,32]	Myocardial	Release in response to ventricular wall stretch and pressure overload	Predicts right ventricular dysfunction, complicated in-hospital course, and 30-day mortality; complements troponin in risk stratification	Influenced by obesity, renal dysfunction, and pre-existing heart failure; non-standardized thresholds across assays
**(B)**				
**Biomarker**	**Predominant Phenotype**	**Mechanism of Release**	**Evidence Level and Potential Application**	**Principal Limitations**
H-FABP [22]	Myocardial	Early release of small cytosolic protein upon myocyte injury (peak ~2 h)	Emerging evidence: rapid early risk stratification; strong prognostic value in normotensive intermediate-risk patients	Assay not universally available; influenced by renal clearance and skeletal muscle injury
GDF-15 [23,36,37,38]	Mixed (myocardial + thromboinflammatory)	Release under oxidative, hypoxic, mechanical, and inflammatory stress	Emerging evidence: independent predictor of complicated 30-day course and long-term mortality	Assay not standardized; rises with age, renal impairment, malignancy, and heart failure
sST2 [24]	Myocardial	Decoy receptor for IL-33 released under mechanical strain and remodeling	Emerging evidence: discriminates intermediate-high from intermediate-low risk (pilot data)	Small single-center cohorts only; no validated thresholds; incremental value over established markers uncertain
Complement activation products/citH3 [11,12,13]	Thromboinflammatory	Complement consumption and NET formation (citrullinated histone H3)	Experimental/research: associated with RV dysfunction; citH3 predicts early mortality in observational studies	Assays not harmonized; reference ranges poorly defined; observational data only
Blood-count indices (NLR, PLR, SII, SIRI, AISI) [39,40,41,42,43]	Thromboinflammatory	Ratio of pro-inflammatory/pro-thrombotic cells to regulatory cells	Emerging evidence: inexpensive, bedside-available; discriminates severity in retrospective cohorts	Values vary with age, sex, and smoking; originally developed in oncology; confounded by infection, malignancy, and corticosteroids
Circulating microRNAs [44,45,46,47,48]	Thromboinflammatory	Secreted in exosomes or protein complexes, resistant to degradation	Experimental/research: moderate diagnostic performance; miR-1233 relatively PE-specific	Technically demanding quantification; hemolysis is a major confounder; no consensus normalizer; small cohorts

Abbreviations: ACS, acute coronary syndrome; BNP, B-type natriuretic peptide; cTnI/cTnT, cardiac troponin I/T; ESC, European Society of Cardiology; NT-proBNP, N-terminal pro-B-type natriuretic peptide; PE, pulmonary embolism; RV, right ventricular. AISI, aggregate index of systemic inflammation; citH3, citrullinated histone H3; GDF-15, growth differentiation factor 15; H-FABP, heart-type fatty acid binding protein; IL-33, interleukin-33; miR-1233, microRNA-1233; NET, neutrophil extracellular trap; NLR, neutrophil-to-lymphocyte ratio; PLR, platelet-to-lymphocyte ratio; SII, systemic immune-inflammation index; SIRI, systemic inflammatory response index; sST2, soluble suppression of tumorigenicity 2.

## Data Availability

No new data were created or analyzed in this study. Data sharing is not applicable to this article.

## References

[1] Zhen K., Tao Y., Xia L., Wang S., Gao Q., Wang D., Chen Z., Meng X., Kang Y., Fan G. (2025). Epidemiology of pulmonary embolism in China, 2021: A nationwide hospital-based study. Lancet Reg. Health West. Pac.

[2] Stein P.D., Matta F., Hughes M.J. (2018). CT Pulmonary Angiography in Young Women. Clin. Appl. Thromb. Hemost..

[3] Barco S., Mahmoudpour S.H., Planquette B., Sanchez O., Konstantinides S.V., Meyer G. (2019). Prognostic value of right ventricular dysfunction or elevated cardiac biomarkers in patients with low-risk pulmonary embolism: A systematic review and meta-analysis. Eur. Heart J..

[4] Faggioni M., Giri J., Glassmoyer L., Kobayashi T. (2025). A rational approach to the treatment of acute pulmonary embolism. Annu. Rev. Med..

[5] Latsios G., Mantzouranis E., Kachrimanidis I., Theofilis P., Dardas S., Stroumpouli E., Aggeli C., Tsioufis C. (2025). Recent advances in risk stratification and treatment of acute pulmonary embolism. World J. Cardiol..

[6] Cucută S., Bădescu M.C., Duca S.T., Chetran A., Cepoi M.R., Ponor C.G., Bobu A.M., Serban I.L., Costache-Enache I.I. (2025). Beyond a single marker: An update on the comprehensive evaluation of right ventricular dysfunction in pulmonary thromboembolism. Life.

[7] Pober J.S., Sessa W.C. (2007). Evolving functions of endothelial cells in inflammation. Nat. Rev. Immunol..

[8] Folco E.J., Mawson T.L., Vromman A., Bernardes-Souza B., Franck G., Persson O., Nakamura M., Newton G., Luscinskas F.W., Libby P. (2018). Neutrophil extracellular traps induce endothelial cell activation and tissue factor production through interleukin 1 alpha and cathepsin G. Arterioscler. Thromb. Vasc. Biol..

[9] Fuchs T.A., Brill A., Wagner D.D. (2012). Neutrophil extracellular trap impact on deep vein thrombosis. Arterioscler. Thromb. Vasc. Biol..

[10] Marcos-Jubilar M., Lecumberri R., Páramo J.A. (2023). Immunothrombosis: Molecular aspects and new therapeutic perspectives. J. Clin. Med..

[11] Rostoff P., Zabczyk M., Natorska J., Undas A. (2024). Complement activation is associated with right ventricular dysfunction and the severity of pulmonary embolism: Links with prothrombotic state. J. Thorac. Dis..

[12] Rostoff P., Zabczyk M., Natorska J. (2024). Enhanced complement consumption and histone H3 citrullination predict disease severity and early mortality risk in non-high-risk pulmonary embolism. Pol. Heart J..

[13] Chen Y., Li X., Lin X., Liang H., Liu X., Zhang X., Zhang Q., Zhou F., Yu C., Lei L. (2022). Complement C5a induces the generation of neutrophil extracellular traps by inhibiting mitochondrial STAT3 to promote the development of arterial thrombosis. Thromb. J..

[14] Förstermann U., Sessa W.C. (2012). Nitric oxide synthases: Regulation and function. Eur. Heart J..

[15] Kline J.A., Watts J., Courtney D., Lee Y.Y., Hwang S. (2014). Severe pulmonary embolism decreases plasma L-arginine. Eur. Respir. J..

[16] Murat M., Pepele M.S., Derya S., Sülü G., Yücel N., Mertoğlu C., Katırcı H.C., Avcu A.B. (2026). Serum Endothelin-1 in Suspected Acute Pulmonary Embolism: A Prospective Study. J. Cardiovasc. Dev. Dis..

[17] Gendron N., Planquette B., Roche A., Chocron R., Helley D., Philippe A., Morange P.E., Gaussem P., Sanchez O., Smadja D.M. (2025). Circulating CD34 positive cells: A new biomarker of residual pulmonary vascular obstruction after pulmonary embolism. Stem Cell Rev. Rep..

[18] Piazza G., Goldhaber S.Z. (2005). The acutely decompensated right ventricle: Pathways for diagnosis and management. Chest.

[19] Konstantinides S.V., Barco S., Lankeit M., Meyer G. (2016). Management of pulmonary embolism: An update. J. Am. Coll. Cardiol..

[20] Konstantinides S.V., Meyer G., Becattini C., Bueno H., Geersing G.J., Harjola V.P., Huisman M.V., Humbert M., Jennings C.S., Jiménez D. (2020). 2019 ESC Guidelines for the diagnosis and management of acute pulmonary embolism developed in collaboration with the European Respiratory Society. Eur. Heart J..

[21] Becattini C., Vedovati M.C., Agnelli G. (2007). Prognostic value of troponins in acute pulmonary embolism: A meta-analysis. Circulation.

[22] Puls M., Dellas C., Lankeit M., Olschewski M., Binder L., Geibel A., Reiner C., Schäfer K., Hasenfuss G., Konstantinides S. (2007). Heart-type fatty acid binding protein permits early risk stratification of pulmonary embolism. Eur. Heart J..

[23] Kempf T., Eden M., Strelau J., Naguib M., Willenbockel C., Tongers J., Heineke J., Kotlarz D., Xu J., Molkentin J.D. (2006). The transforming growth factor beta superfamily member growth differentiation factor 15 protects the heart from ischaemia and reperfusion injury. Circ. Res..

[24] Wang J., Liu Z., Jiao Y., Cheng Y., Li J. (2025). The value of sST2 in risk stratification and short-term prognosis of acute pulmonary embolism: A pilot study focusing on intermediate-risk subgroups. Front. Cardiovasc. Med..

[25] Creager M.A., Barnes G.D., Giri J., Mukherjee D., Jones W.S., Burnett A.E., Carman T., Casanegra A.I., Castellucci L.A., Clark S.M. (2026). 2026 AHA/ACC/ACCP/ACEP/CHEST/SCAI/SHM/SIR/SVM/SVN Guideline for the Evaluation and Management of Acute Pulmonary Embolism in Adults: A Report of the American College of Cardiology/American Heart Association Joint Committee on Clinical Practice Guidelines. Circulation.

[26] Wells P.S., Anderson D.R., Rodger M., Ginsberg J.S., Kearon C., Gent M., Turpie A.G., Bormanis J., Weitz J., Chamberlain M. (2000). Derivation of a simple clinical model to categorize patients’ probability of pulmonary embolism: Increasing the models’ utility with the SimpliRED D-dimer. Thromb. Haemost..

[27] Righini M., Van Es J., Den Exter P.L., Roy P.M., Verschuren F., Ghuysen A., Rutschmann O.T., Sanchez O., Jaffrelot M., Trinh-Duc A. (2014). Age-adjusted D-dimer cutoff levels to rule out pulmonary embolism: The ADJUST-PE study. JAMA.

[28] Klok F.A., Cohn D.M., Middeldorp S., Schut A., Büller H.R., van Dissel J.T., Huisman M.V. (2008). Use of clinical prediction rules and D-dimer to rule out pulmonary embolism in patients with cancer. Br. J. Haematol..

[29] Douma R.A., Le Gal G., Söhne M., Righini M., Kamphuisen P.W., Perrier A., Kruip M.J., Bounameaux H., Büller H.R., Roy P.M. (2010). Potential of an age adjusted D-dimer cut-off value to improve the exclusion of pulmonary embolism in older patients: A retrospective analysis of three large cohorts. BMJ.

[30] Jiménez D., Aujesky D., Moores L., Gómez V., Lobo J.L., Uresandi F., Otero R., Monreal M., Muriel A., Yusen R.D. (2011). Combinations of prognostic tools for identification of high-risk normotensive patients with acute symptomatic pulmonary embolism.. Thorax.

[31] Bova C., Sanchez O., Prandoni P., Lankeit M., Konstantinides S., Vanni S., Jiménez D. (2014). Identification of intermediate-risk patients with acute symptomatic pulmonary embolism. Eur. Respir. J..

[32] Hanyu Y., Hoshino M., Usui E., Sugiyama T., Kanaji Y., Hada M., Nagamine T., Nogami K., Ueno H., Yonetsu T. (2025). Clinical Significance of Coronary Flow Reserve Improvement After Elective Percutaneous Coronary Intervention. Catheter. Cardiovasc. Interv..

[33] Klok F.A., Mos I.C.M., Huisman M.V. (2008). Brain-type natriuretic peptide levels in the prediction of adverse outcome in patients with pulmonary embolism: A systematic review and meta-analysis. Am. J. Respir. Crit. Care Med..

[34] Vuilleumier N., Le Gal G., Verschuren F., Perrier A., Bounameaux H., Turck N., Sanchez J.C., Mensi N., Perneger T., Hochstrasser D. (2009). Cardiac biomarkers for risk stratification in non-massive pulmonary embolism: A multicentre prospective study. J. Thromb. Haemost..

[35] Clerico A., Franzini M., Masotti S., Prontera C., Passino C. (2014). State of the art of BNP and NT-proBNP immunoassays: The CardioOrmoCheck study. Clin. Chim. Acta.

[36] Lankeit M., Kempf T., Dellas C., Cuny M., Tapken H., Peter T., Olschewski M., Konstantinides S., Wollert K.C. (2008). Growth differentiation factor 15 for prognostic assessment of patients with acute pulmonary embolism. Am. J. Respir. Crit. Care Med..

[37] Skowrońska  M., Skrzyńska M., Machowski M., Bartoszewicz Z., Paczyńska M. (2020). Plasma growth differentiation factor 15 levels for predicting serious adverse events and bleeding in acute pulmonary embolism: A prospective observational study. Pol Arch Intern Med.

[38] Wollert K.C., Kempf T., Wallentin L. (2017). Growth differentiation factor 15 as a biomarker in cardiovascular disease. Clin. Chem..

[39] Uslu M.F., Yılmaz M., Ateşçelik M., Atilgan F.A. (2024). The utility of the systemic immune inflammation and systemic inflammation response indices in suspected intermediate-risk pulmonary embolism. Croat. Med. J..

[40] Siddiqui F., Tafur A., Hussain M., García-Ortega A., Darki A., Fareed J., González Jiménez D., Bikdeli B., Galeano-Valle F., Fernández-Reyes J.L. (2024). The prognostic value of blood cellular indices in pulmonary embolism. Am. J. Hematol..

[41] Hu B., Yang X.R., Xu Y., Sun Y.F., Sun X., Qiu S.J., Yang G.H., Dai Z., Zhou J., Fan J. (2014). Systemic immune-inflammation index predicts prognosis of patients after curative resection for hepatocellular carcinoma. Clin. Cancer Res..

[42] Qi J., Li Q., Li Q., Wang X., Li H., Zhang X., Zhang H., Zhang L. (2016). The systemic inflammation response index predicts prognosis in patients with advanced non-small cell lung cancer. Oncotarget.

[43] Zhang K., Hua Y., Wang D., Chen L., Wu C., Chen Z., Liu L., Chen H. (2019). Systemic immune-inflammation index predicts prognosis of patients with advanced pancreatic cancer. J. Transl. Med..

[44] Sobrero M., Montecucco F., Carbone F. (2022). Circulating microRNAs for diagnosis of acute pulmonary embolism: Still a long way to go. BioMed. Res. Int..

[45] Xiang Q., Zhang H., Wang Z., Liu Z., Xie Q., Hu K., Zhang Z., Mu G., Ma L., Jiang J. (2019). The predictive value of circulating microRNAs for venous thromboembolism diagnosis: A systematic review and diagnostic meta-analysis. Thromb. Res..

[46] Kessler T., Erdmann J., Vilne B., Bruse P., Kurowski V., Diemert P., Schunkert H., Sager H.B. (2016). Serum microRNA-1233 is a specific biomarker for diagnosing acute pulmonary embolism. J. Transl. Med..

[47] Deng H.Y., Li G., Luo J., Wang Z., Yang X., Lin Y.D., Liu L. (2016). MicroRNAs are novel non-invasive diagnostic biomarkers for pulmonary embolism: A meta-analysis. J. Thorac. Dis..

[48] Gareev I., Pavlov V., Du W., Yang B. (2023). miRNAs and their role in venous thromboembolic complications. Diagnostics.

[49] Dellas C., Puls M., Lankeit M., Schäfer K., Cuny M., Berner M., Hasenfuss G., Konstantinides S. (2010). Elevated heart-type fatty acid-binding protein levels on admission predict an adverse outcome in normotensive patients with acute pulmonary embolism. J. Am. Coll. Cardiol..

[50] Tschepe M., Seeber V., Lankeit M., Tello K., Ghofrani H.A., Schwaab J., Münzel T., Konstantinides S. (2014). A novel H-FABP assay and a fast prognostic score for risk assessment of normotensive pulmonary embolism. Thromb. Haemost..

[51] Ruan L.B., He L., Zhao S., Zhu P., Li W.Y. (2014). Prognostic value of plasma heart-type fatty acid-binding protein in patients with acute pulmonary embolism. Chest.

[52] Bajaj A., Rathor P., Sehgal V., Shetty A., Kabak B., Hosur S. (2015). Risk stratification in acute pulmonary embolism with heart-type fatty acid-binding protein: A meta-analysis. J. Crit. Care.

[53] Lankeit M., Dellas C., Benz V., Hasenfuß G., Konstantinides S. (2013). The predictive value of heart-type fatty acid-binding protein is independent from symptom duration in normotensive patients with pulmonary embolism. Thromb. Res..

[54] Lankeit M., Dellas C., Kramm T., Kamp J., Vazir A., Nagel C., Ghofrani H.A., Mayer E., Weidenhammer M., Konstantinides S. (2008). Heart-type fatty acid-binding protein for risk assessment of chronic thromboembolic pulmonary hypertension. Eur. Respir. J..

[55] Ferrari E., Moceri P., Benichou A., Baudouy M. (2012). Timing of troponin I measurement in pulmonary embolism. Heart.

[56] Subba H., McKenna A., Gilboy J., Gelman J., Evans J., Smith A., Kerney J., Wirth J.A. (2026). Prospective evaluation of serial biomarkers in patients with intermediate-high risk acute pulmonary embolism: A single-center proof-of-concept study. Pulm. Circ..

[57] Becattini C., Vedovati M.C., Colombo S., Vanni S., Abrignani M.G., Scardovi A.B., Marrazzo A., Borselli M., Barchetti M., Fabbri A. (2024). Identification of hemodynamically stable patients with acute pulmonary embolism at high risk for death: External validation of different models. J. Thromb. Haemost..

[58] Buonacera A., Stancanelli B., Giarratana D., Malatino L. (2025). Evidence for the Interplay between Inflammation and Clotting System in the Pathogenetic Chain of Pulmonary Embolism: A Potential Therapeutic Target to Prevent Multi-organ Failure and Residual Thrombotic Risk. Curr. Vasc. Pharmacol..

